# The Relationship Between Physical Activity Levels and Manual Dexterity in Adults Over 60 Years Old

**DOI:** 10.1093/geroni/igaa057.1604

**Published:** 2020-12-16

**Authors:** Naomi Kim, Rachael McGraw, Katie Thralls

**Affiliations:** 1 Seattle Pacific University, Spokane, Washington, United States; 2 Seattle Pacific University, Seattle, Washington, United States

## Abstract

During aging there is a natural physiological decline that contributes to a loss of function needed for activities of daily living to maintain independence and high quality of life. Physical function needed for independence includes gross motor function (e.g., lower body strength for standing) and fine motor function (e.g., manual dexterity for dressing). Physical activity (PA) has shown to maintain fitness, such as muscular strength, to delay loss in gross motor function. However, there is limited research on the association between PA and fine motor function. The purpose of this study was to examine the relationship between meeting national Physical Activity Guidelines (PAG; >150 min./wk.) and manual dexterity in older adults (>60 years). Participants (N=45, Mean age = 80.2±8.2 years) completed an interview-assisted self-report of their PA level and an objectively measured manual dexterity assessment (i.e., Purdue Pegboard Test (PPT)). The PPT included four fine motor skill assessments. For all four PPT’s, Analysis of Variance (ANOVA) tests showed a significant main effect for PA level, a main effect for age, and an interaction effect (PA*age) on manual dexterity for all PPTs (ps<0.05). Follow-up comparisons showed a significant main effect for PA level on manual dexterity for the older group (>80yrs; ps<0.05), and not for the younger group (ps>0.05). Pearson’s r correlations showed significant moderate-positive correlations between activity level (min./wk.) and PPTs scores (r=0.45– 0.50; ps<0.005). These findings suggest that meeting PAG may be a preventative strategy to attenuate aging declines in manual dexterity to maintain hand function and independence.

